# Using decision analysis to support implementation planning in research and practice

**DOI:** 10.1186/s43058-022-00330-1

**Published:** 2022-07-30

**Authors:** Natalie Riva Smith, Kathleen E. Knocke, Kristen Hassmiller Lich

**Affiliations:** 1 Department of Social and Behavioral Sciences, Harvard TH Chan School of Public Health, Harvard University, Boston, MA 02115 USA; 2 Department of Health Policy and Management, Gillings School of Global Public Health, UNC Chapel Hill, Chapel Hill, USA

**Keywords:** Implementation science, Cost and cost analysis, Economic evaluation, Decision support techniques, Decision-making

## Abstract

**Background:**

The process of implementing evidence-based interventions, programs, and policies is difficult and complex. Planning for implementation is critical and likely plays a key role in the long-term impact and sustainability of interventions in practice. However, implementation planning is also difficult. Implementors must choose what to implement and how best to implement it, and each choice has costs and consequences to consider. As a step towards supporting structured and organized implementation planning, we advocate for increased use of decision analysis.

**Main text:**

When applied to implementation planning, decision analysis guides users to explicitly define the problem of interest, outline different plans (e.g., interventions/actions, implementation strategies, timelines), and assess the potential outcomes under each alternative in their context. We ground our discussion of decision analysis in the PROACTIVE framework, which guides teams through key steps in decision analyses. This framework includes three phases: (1) definition of the decision problems and overall objectives with purposeful stakeholder engagement, (2) identification and comparison of different alternatives, and (3) synthesis of information on each alternative, incorporating uncertainty. We present three examples to illustrate the breadth of relevant decision analysis approaches to implementation planning.

**Conclusion:**

To further the use of decision analysis for implementation planning, we suggest areas for future research and practice: embrace model thinking; build the business case for decision analysis; identify when, how, and for whom decision analysis is more or less useful; improve reporting and transparency of cost data; and increase collaborative opportunities and training.

Contributions to the literature

- We introduce decision analysis for implementation planning as a way to overcome common challenges faced in the planning process, such as uncertainty about how to select interventions or implementation strategies in a given context or reconciling competing objectives among stakeholders.
- We discuss the PROACTIVE framework, which describes three broad phases of decision analysis and guides users through explicitly defining the problem of interest, outlining different implementation plans, assessing the potential outcomes of each, and considering those outcomes in context.
- We provide key areas for future research to consider on the path towards advancing decision analysis to support implementation planning.

## Background

Early implementation involves many choices [1–3]. These choices involve questions such as what intervention or evidence-based program (EBP) should be pursued for a given health issue of interest, or, if the intervention is already selected, what implementation strategies will best support success. Combined intervention/implementation strategy packages might also be considered. These choices are difficult because there are ever-increasing options for interventions and implementation strategies, and early decisions likely influence subsequent choices or future implementation plans (e.g., adding additional interventions in the future, when resources allow). Insurmountable barriers to implementing an intervention might arise that are unique to a given context, requiring planners to reevaluate their intervention choice, and contextually appropriate implementation strategies are challenging to select [4, 5].

In addition to considering the likely effectiveness of different intervention and/or implementation strategy combinations (hereafter referred to as decision “alternatives”), implementors also need to consider the cost implications of each alternative and determine whether they are feasible. Cost considerations—such as how much an intervention and strategy combination might cost, what the timing of those costs is, and who is responsible for those costs—have emerged as key drivers of implementation success [6–11]. During planning, implementors may ask questions such as “What might be the relative costs of implementation strategy A versus B, compared to the expected consequences of each?” or “How much staff time might be needed to implement this intervention with fidelity?” These questions about the costs and consequences of different alternatives, also called economic evaluations, can be used to help analyze trade-offs between different implementation plans [12, 13]. Economic evaluation can also help implementors plan for the financial realities of implementation and facilitate buy-in from investors or stakeholders [9, 14].

Additional decision objectives may also be important, with precise objectives being context specific. Sometimes, equity impacts are a priority, other times mitigating potential risks (e.g., harm, failing to be cost-neutral) may be important. One available resource for implementation planning that accounts for the variety of objectives and considerations of implementation is the RE-AIM project planning tool, which includes questions about the expected effects of a program, its required resources, and staff capacity [15]. Another resource, the Implementation Research Logic Model, guides users to think through potential implementation strategies and scenarios based on known parameters [16]. While these kinds of planning tools are extremely valuable contributions to implementation science, they are limited in that, for example, they presume a specific intervention is already chosen or provide minimal guidance on how to compare alternative implementation plans.

The complexity of implementation planning also makes existing tools limited. Thinking through implementation planning involves many characteristics that make decisions difficult: long-time horizons (requiring action up-front, though benefits may not be realized until later), the involvement of many different stakeholders with different values and preferences, uncertainty in the possible outcomes under different alternatives, and interconnected decisions [17–19]. This complexity makes systematic decision-making difficult, particularly because individuals tend to rely on simplifications or heuristics to make decisions in the face of complexity, leading to biased, inconsistent, or even harmful decisions [17, 20–27].

Despite the importance and complexity of implementation planning, it has received relatively little attention in the literature [3, 15, 28] and approaches are needed to help structure the planning process and weigh different alternatives. An ideal approach would be flexible enough to meet a range of planning questions and integrate multiple considerations to help answer complex questions that arise during the planning process. We believe that decision analysis, a widely used and flexible process to systematically approach decision-making, offers just that. In this paper, we discuss the decision analysis approach, with particular attention to its relevance for implementation planning questions.

## What is decision analysis?

Decision analysis is a systematic way to assess various aspects of complex problems under uncertain conditions, with the goal of helping decision-makers choose the course of action (i.e., alternative) that best aligns with their objectives, considering their own context [17, 18, 29–33]. Applied to implementation planning questions, decision analysis aims to provide structure to the planning process by ensuring that assumptions, possible decision alternatives, available research evidence, and objectives for implementation are laid out systematically and explicitly. Within public health and healthcare, readers may be familiar with patient-level decision analysis (i.e., operationalized in clinical decision aids) that aims to help patients choose treatments that best align with their own care needs and preferences [34] or, with larger, national-level decision analytic approaches such as those used in the UK to structure healthcare reimbursement decisions [35]. In the context of this paper, we take a general view and consider decision-makers to be relevant stakeholders engaged with the implementation planning processes (e.g., those who are making adoption decisions, as well as those who can choose to support/resist decisions) [36] and decision problems to be implementation planning questions (typically about intervention or implementation strategy choices).

We structure our discussion using the PROACTIVE framework as a guide. PROACTIVE is a foundational framework for decision analysis in health introduced by Hunink and colleagues [17], which draws on work from Keeney and colleagues in operations research [18, 19]. PROACTIVE offers a comprehensive overview of established steps in decision analysis while allowing for flexibility and iteration within each [17]. The framework conceptualizes decision analysis as a process spanning three phases through which (1) the decision problem and overall objectives are defined, (2) different alternatives are compared, and (3) information on each alternative is synthesized [17]. Overall, this framework provides a clear way to understand the full process of decision analysis, without being overly prescriptive about which specific methods are used throughout.

Throughout our discussion, we use three examples of decision analysis for implementation planning to illustrate how various steps of the PROACTIVE framework can be operationalized (Table 1). Examples were selected for their heterogenous approaches and focuses and to showcase the variety of ways that decision analysis could be approached in implementation planning efforts: selecting childhood maltreatment EBPs (Cruden et al.), improving the reach of EBPs for mental health (Zimmerman et al.), and improving rates of colorectal cancer screening (Hassmiller Lich et al.) [37–39].

**Table 1:** Three examples of decision analysis processes for implementation planning

**Step in the PROACTIVE framework**	**Cruden et al.** [37]	**Zimmerman et al.** [38]	**Hassmiller Lich et al.** [39]
Phase 1			
P: Define the problem	Engaged stakeholders to identify a specific problem related to child maltreatment that they felt needed to be solved	– Paper focused on limited EBP for PTSD reach	– Paper focused on low rates of colorectal cancer screening and major disparities
R: Reframe from other perspectives and understand objectives	Diverse stakeholders included in entire process (e.g., local health departments, state legislators, state employees, and non-profit administrators)	Engaged stakeholders from a variety of roles, including both clinic leadership and frontline staff members	– Study was explicitly focused on estimating cost-effectiveness from the perspective of Medicaid
O: Focus on the unifying objectives	Stakeholders prioritized child neglect and focused on prevention, and the team and stakeholders worked together to clarify the criteria that would be used to evaluate EBPs	Increase uptake of EBPs for PTSD and depression within the VA while acknowledging existing system constraints (e.g., staffing)	Stated objectives of analysis were to compare the impact and cost-effectiveness of colorectal cancer screening interventions in North Carolina and their potential to reduce disparities
Phase 2			
A: Collate alternatives	Research team compiled a list of 7 EBPs for comparison in an MCDA, three were prioritized for final evaluation	Contexts were interested in how to revise clinic procedures, workflows, or roles to increase the reach of EBPs. Stakeholder engagement indicated many possible ways to do this, and priority ranking was used to identify the top two for further evaluation (Table 3b in text)	Interventions defined via literature review and a series of interviews with decision-makers and local stakeholders
C: Model consequences	Scored each EBP on criteria using information from published literature	Used system dynamics modeling to simulate how different implementation plans would affect EBP uptake within a specific context, using equations that represented the flows and accumulations of patients based on system structure	Quantitative microsimulation model was used to estimate the potential effects and costs of each screening intervention
How costs were incorporated	Two criteria explicitly related to costs were included: benefits minus cost, and per participant per year; chances benefits will exceed costs. Other criteria also included a focus on resources required to implement, including the total timeframe to deliver the EBP, requirements for education/training of EBP deliverer, and whether ongoing support was available to facilitate implementation. Information on all was collected from the existing literature	The cost per implementation plan was not specifically included; rather, each implementation plan was subject to the existing resource and staffing constraints of local contexts	Costs to implement each intervention were explicitly enumerated from the state’s perspective, with components clearly listed and sourced (Table 2 in text)
T: Identify trade-offs	Stakeholders engaged to develop weights for each criterion that described the relative importance of each criterion	– No explicit preferences or values work was incorporated	– No explicit preferences or values work was incorporated (e.g., to identify how Medicaid decision-makers might weigh costs versus effects of each policy)
Phase 3			
I: Integrate evidence to calculate expected value	A summary score was calculated for each EBP by combining criteria scores and weights (steps C and T)	Visualizing trends in EBP uptake over time allowed stakeholders to see how (and when) different implementation plans would impact outcomes	Incremental cost-effectiveness ratios were calculated and trade-offs in cost and life years up to date were shown visually using a cost-effectiveness efficiency frontier
V: Optimize expected value	Process not focused on identifying a global optimal choice, but rather presenting scored options for stakeholders to use and identify their own optimal path accounting for personal values and preferences (by, for example, modifying weights)	– Process focused on building the model and simulating different implementation plans	– No decision was made as the paper’s primary focus was to generate evidence for decision-makers (shown via cost-effectiveness ratios and the cost-effectiveness efficiency frontier). The most cost-effective options were mentioned
E: Explore assumptions and uncertainty	Sensitivity analyses used average weights (rather than individualized weights for each decision-maker) to calculate summary scores	Model was calibrated using historical data, sensitivity analyses and stakeholder review used to validate model, and sensitivity analyses were performed using different implementation decisions	Model was extremely large and probabilistic analyses not reported, and model was tested using code review, extreme-value testing, behavior-reproduction testing, and review with content experts

*Notes*: Alignment with PROACTIVE framework performed by the authorship team based on the information reported in published work. “–” indicates that the step of the PROACTIVE framework was not an obvious focus of the published paper, illustrating how not all steps need be employed for any given problem

*EBP* evidence-based program, *MCDA* multi-criteria decision analysis, *PTSD* post-traumatic stress disorder, *VA* Veterans Health Administration

### Phase 1: PRO—defining problem and objectives

#### P: Defining the problem

It is critical to understand the precise nature of the problem at hand before making decisions. This often involves understanding the “natural history” of the problem—what is happening over time and what might happen if we take no action? In implementation science, this may be health problems we seek to address (e.g., disparities in blood pressure control) or low uptake of EBPs we have already decided to support (e.g., for depression treatment among veterans). Understanding the determinants of health is also important, including system structure flaws that need to be addressed as well as what barriers or facilitators to implementation exist—both broadly and specific to considered interventions. Here, it is critical that focal problems be understood in the context they will be addressed, asking and answering the question “Why does this problem persist here and now?”.

#### R: Reframe from other perspectives and understand objectives

Especially with complex issues in implementation that are not constrained to a single industry, discipline, or sector, multiple perspectives are critical. The problem, and what each stakeholder hopes to accomplish, might look quite different. It may take discussion to develop a shared vocabulary and understanding before the core problem, objectives, and the most critical determinants of the problem become clear. Even when prioritized decision objectives overlap, there may remain varied preferences across perspectives. Differences in objectives must be understood and acknowledged, and interconnections and commonalities highlighted to support cross-stakeholder change initiatives.

#### O: Focus on the unifying objective(s)

The unifying objectives of stakeholders—what a group can unite behind in a change initiative—typically become apparent as the problem is discussed and reframed. Objectives can be competing (e.g., access and system cost), and their priority could differ by stakeholders and across contexts. They might also be different over time (e.g., access matters first, then quality of care). Objectives may include meeting certain benchmarks for standard implementation outcomes such as fidelity or sustainability [40] or maximizing health benefits. Costs to implement and sustain the intervention are also often relevant; for example, stakeholders may want to keep costs to a minimum or within a prespecified range (potentially because of grant restrictions or a budget). Other objectives such as the potential harms of a given implementation plan or impacts on health equity may be relevant to stakeholders. Still, other objectives might be shaped by organizations’ missions. The goal in this stage of the process is to identify the set of objectives that stakeholders, as a group, agree upon in order to frame the next steps in the decision analysis process.

These first three steps are interconnected—and as such, those involved in decision analysis processes should acknowledge this and ensure these steps are flexible enough to allow for feedback and learning. The importance of stakeholder engagement in these steps cannot be overstated. Without the purposeful and meaningful engagement of relevant stakeholder groups, a problem statement and objectives might be settled on that will later be resisted—threatening sustainability—due to insufficient support or misalignment with the true structure of the system producing problematic outcomes [23]. Traditional qualitative work (e.g., key informant interviews, focus groups) or quantitative work (e.g., surveys sent to stakeholders) can be leveraged here, and we also suggest that researchers consider methods from the field of systems science that have developed engagement processes specifically designed to facilitate a shared understanding of a problem and objectives through engaging diverse stakeholders in structured systems thinking processes—particularly valuable when motivating problems are complex [41, 42].

The case studies each approached these steps somewhat differently. First, Cruden engaged a group of stakeholders to clarify their definition of childhood maltreatment and identify objectives (referred to as criteria in their work) by which to evaluate different EBPs, such as program familiarity, strength of evidence base, or resource availability [37]. Conversely, Zimmerman et al. began their work with a clear problem and objectives (limited reach of EBPs for mental health, increasing scheduled and completed appointments) and spent time conducting qualitative work with stakeholders to understand the components of the system under study and how perspectives on the system differed between stakeholders [38]. Finally, Hassmiller Lich had an a priori problem definition and objectives (low rates of colorectal cancer screening, understanding the cost-effectiveness of different interventions from Medicaid’s perspective) [39].

### Phase 2: ACT—comparing different alternatives

#### A: Consider relevant alternatives (interventions and/or implementation strategies)

It is critical to know the range of what alternatives are possible before decisions are made—including doing nothing (a decision itself). Information on possible interventions can come from a variety of sources, including evidence searches or consults with stakeholders and experts. Online repositories such as the National Cancer Institute’s Evidence-Based Cancer Control Programs listing can help identify interventions that align with different focus areas (e.g., HPV vaccination, tobacco control) or populations (e.g., rural adults, schoolchildren) [43]. Tools such as the CFIR-ERIC matching tool can help narrow the possible universe of implementation strategies based on context- or intervention-specific barriers [5]. It is also important in this stage to understand what is already in place in the community that can be built on (and not duplicated) and what resources are available to be leveraged.

#### C: Understand the possible consequences of each alternative

The next stage involves considering the consequences of each alternative—aligned with the objectives of interest. For example, how might the intervention impact health outcomes? What is the cost of proposed implementation strategies? Are there additional consequences that might bolster or undermine intended effects? Who might react to changes the intervention creates—and how will those reactions impact the objectives of interest?

There is a wide range of methods that can be used to understand consequences. As is true with all research, the optimal method(s) will be based on the objectives of implementation planning, available research resources and capacity, and the specific questions that need to be answered. One simple approach is to collate existing literature, reports, or evaluations on the likely consequences under each alternative. Quantitative and computational simulation modeling can be undertaken as well. Decision analysis experts including Briggs et al. [44] and Marshall et al. [45, 46] provide quality overviews of modeling methods and how they align with different questions of interest, along with references for additional reading. The wide range of potential methods available allows those with differing questions, expertise, resources, and/or decision urgency to engage effectively. For example, queuing and discrete event modeling are typically employed when questions focus on service delivery systems or waiting times [47, 48]. Agent-based modeling, on the other hand, can simulate interacting individuals in geographically explicit contexts, making it particularly useful when implementation planning depends on social network effects or geographic considerations [49].

One modeling method we wish to draw particular attention to is system dynamics modeling, which focuses on conceptualizing relationships between variables over time, identifying feedback loops between them, and modeling how the system responds to changes over time [50, 51]. These models simulate how accumulating quantities (say, the number of individuals who are up-to-date with colorectal cancer screening) change over time. As part of the broader field of systems science, system dynamics modeling has an explicit focus on and ability to simulate the elements of complexity present in implementation science work [52–57]. For example, time dynamics and delays during the implementation process are typical (e.g., change takes time, costs accrue quickly while health benefits accrue more slowly, data and system feedback is often delayed in its availability), and feedback loops can exist among relevant implementation considerations (i.e., when something in the system responds to earlier changes, either reinforcing or counteracting earlier changes—in both desired and undesired ways). When these types of characteristics are present, breaking down complex systems into pieces and simplifying assumptions allows for studying individual pathways, but evaluating pathways independently of the broader system can lead to major missed opportunities or even exacerbation of the problem that motivated intervention [23, 58, 59]. These qualities make system dynamics a natural fit for implementation science work [38, 52, 53, 60].

#### T: Identify and estimate trade-offs (preferences and values)

Once an understanding of the potential consequences has been established, the trade-offs between decision alternatives can be examined. For example, one implementation strategy may cost more but improve implementation outcomes more than a cheaper implementation strategy, or one implementation plan might be expected to be less effective overall but reduce health inequities. These kinds of results raise important trade-offs that decision-makers must acknowledge and consider when making decisions. To plan in the face of trade-offs requires an understanding of what decision-makers prefer and value (e.g., is it more important to improve fidelity or feasibility?), all of which may be context-specific [33, 61].

A major consideration when incorporating values and preferences into implementation planning is to consider whose values and preferences are being used [33, 62]. For example, a clinic could use the values of their patient population, or front-line providers, or administration when weighing trade-offs between alternatives. In some situations, preferences and values may align across stakeholders, and in other cases, they may not. Mixed-methods approaches can help capture how different perspectives and contexts relate to trade-offs in costs and consequences [61].

One particular method of use here is discrete choice experiments (DCEs), which focus on quantifying the relative importance of different aspects of alternatives [63]. Applied to implementation planning, DCEs could be used to gather information from stakeholders on which interventions or implementation strategies they prefer [63], or identify trade-offs between different aspects of implementation plans (e.g., costs, effects, feasibility), allowing for a more comprehensive assessment of the value of different implementation plans.

Cruden et al. identified seven candidate EBPs for child maltreatment prevention from a best practices repository, including three in the final analyses; each intervention was scored on each stakeholder-identified criteria using published literature, and weights were used to capture preferences for different criteria [37]. In Zimmerman’s work, stakeholders suggested many potential implementation plans, and the team worked to prioritize their top two for further analysis (implicitly incorporating preferences) [38]. The potential effects of these plans were quantitatively compared using a system dynamics simulation model, developed and calibrated with local data (see Fig. 2 in text for a visual of the system structure) [38]. Finally, Hassmiller Lich compared four interventions, selected via literature reviews and stakeholder interviews, using a large individual-based simulation model that estimated each intervention’s likely costs (in dollars) and effects (in years of life up-to-date on screening) [39].

### Phase 3: IVE—synthesizing information on each alternative

#### I: Integrate evidence on the likely consequences given identified preferences and values

It may become clear based on anticipated consequences of alternatives and preferences/values what the “best” decision is for a specific context. Other times, it may not be. In the latter case, using a formal “objective function” that integrates objectives through weighting each component is useful. For example, quantifying the cost per additional patient reached or the net benefit of a decision alternative can integrate costs and health benefits. However, if stakeholders with different perspectives disagree on weights, it may be more useful to present information about expected outcomes for each component (i.e., valued outcome) in a tabular format (often called a “balance sheet,” “values framework,” or “performance matrix” [62, 64]).

#### V: Optimize the expected value

In traditional decision analysis, there are employable rules for making decisions—for example, by choosing the alternative that optimizes an objective or an objective function value or by choosing the alternative that has the smallest chance of poor outcomes. If stakeholders cannot agree on weights for each decision outcome, decision analysts might ask each to select their top 3 alternatives once outcome estimates are available or make their own weights/preferences explicit before seeing outcome estimates. In either case, a process will also be needed for determining which alternative is selected once all stakeholder selections are made (e.g., will each stakeholder get an equal vote, how will ties be broken). These decision rules should be decided through discussion with all involved stakeholders before alternatives’ scores are quantified and analyzed to minimize biases and conflicts.

#### E: Explore assumptions and evaluate uncertainty

Even when significant investment is made to reflect the local context in the decision analysis process when estimating potential outcomes, uncertainty will always exist. Decision analysis findings can only reflect what is known (not what is unknown), and no model will ever precisely anticipate what could be [33]. Presenting single estimates of the consequences of alternatives can mask this uncertainty and potentially mislead decision-makers.

Once expected (best-guess) results are estimated, a cornerstone of decision analysis is exploring how consequences and trade-offs change under different assumptions. This can help decision-makers understand the likelihood of different outcomes based on the uncertainty in different aspects of the decision problem. Some uncertainties may be driven by questions about what could happen (e.g., how much an intervention would change health outcomes, or how effective an implementation strategy might be in promoting adherence). Sometimes uncertainty could be in preferences (e.g., how much is one consequence valued compared to another). Various analytic approaches can be used to explore this uncertainty and inform learning and planning. Across these approaches, we note that uncertainty can only be explored insofar as it is a known source of uncertainty, and engaging with diverse evidence bases, data, and stakeholders improves the chances that analytic models better characterize what is known and anticipate potential unknowns.

Optimization analyses help answer questions such as, “Across different implementation plans, what is the optimal course of action given specific constraints (e.g., hours available for specific types of workers, maximum allowable wait times)?” [30]. Here, “optimal” is defined by decision-makers and often involves minimizing implementation cost or maximizing impact while not violating specific constraints [30]. Factoring in finite resource constraints can be helpful when considering the risks and barriers related to specific implementation plans. For example, consider a clinic wanting to use grant funds to implement a new intervention, but that cannot afford the salary of a new provider. Given the varying costs/impacts for different providers (e.g., physicians, nurse practitioners) to perform implementation and intervention tasks, the clinic could identify the optimal provider (or provider mix) to undertake required tasks while avoiding hiring an additional provider.

**Fig. 1:**
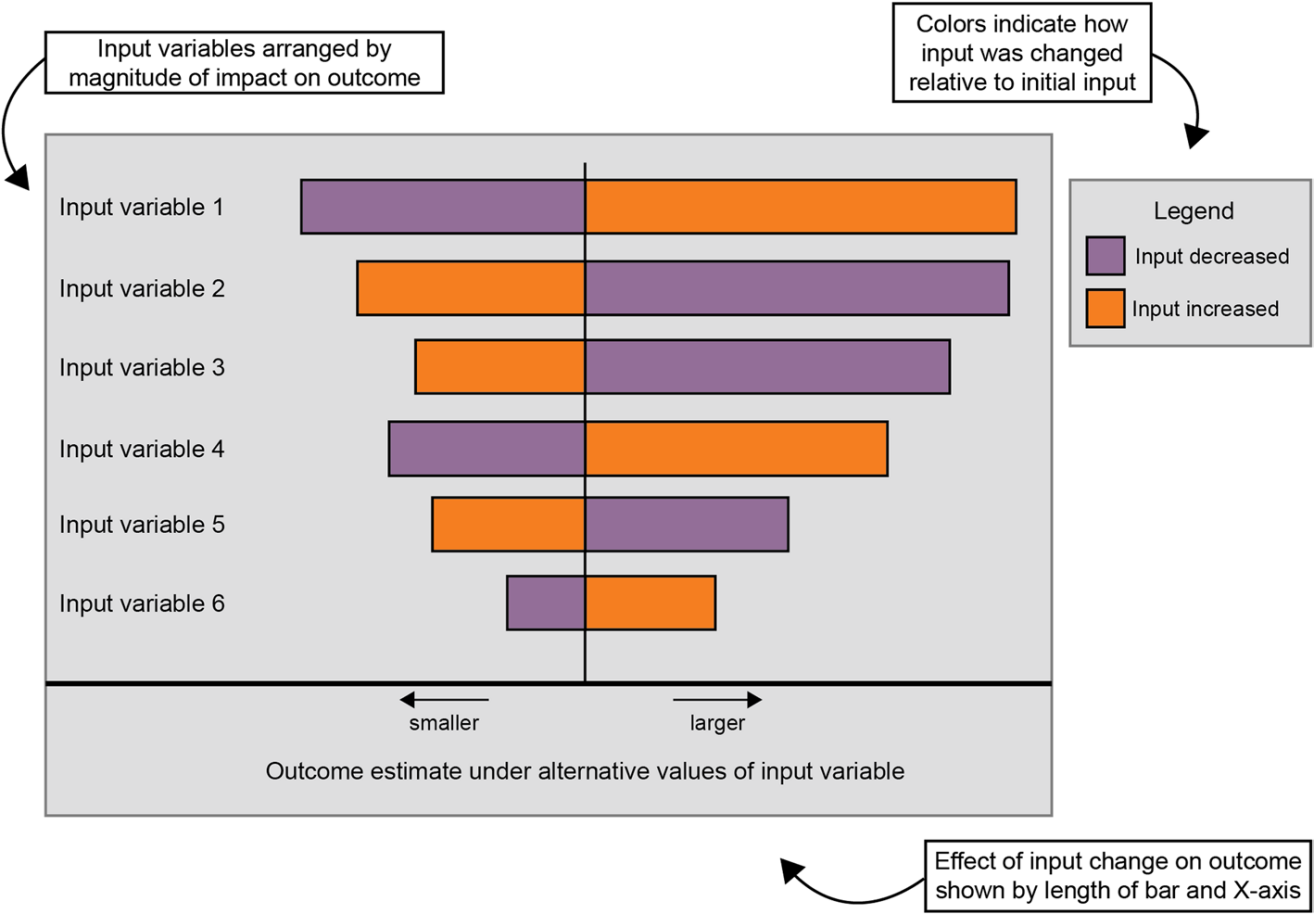
Annotated example of a tornado plot displaying results of single-variable sensitivity analyses. *Notes*: In this figure, variable 1 has the greatest impact on the target outcome as depicted in the figure by the length of the bars and thus may warrant particular attention during the planning process and formal implementation

Sensitivity analyses help identify which decision analysis model input values have the greatest leverage on outcomes [29, 65]. Single variable sensitivity analyses involve changing single inputs and recording the outcomes (i.e., deterministic sensitivity analyses). The results of these analyses can be displayed in a “tornado plot” such as Fig. 1, which clearly communicates which parameters have the potential to affect the focal outcome the most. This kind of information can refine implementation planning by helping planners understand where more (or less) attention and effort should be focused to achieve desired outcomes. Multiple inputs can also be manipulated at once to get a sense of how different scenarios impact outcomes (for example, the lowest or highest values for all inputs). Threshold conditions can also be assessed with sensitivity analyses, for example, to learn the conditions under which costs remain below a certain benchmark, or to estimate required resources to ensure a certain level of effectiveness.

Probabilistic methods can be used to quantify how uncertainty in inputs translates into holistic decision uncertainty, asking such questions as “Are conclusions about a given intervention’s benefits relative to its costs robust to simultaneous realistic variation in inputs?” [29]. While a flavor of this type of analysis can be conducted using multivariable sensitivity analyses, this analytic approach typically involves the simulation of many combinations of inputs using probability distributions to generate a diverse, representative range of possible outcomes. This type of analysis informs decision-makers how varied outcomes might be, given plausible uncertainty in model parameters.

Uncertainty analyses can also be used to drive future research directions. Deterministic sensitivity analyses can help decision-makers pinpoint specific model uncertainties that, if addressed through further research, could improve decision-making when current uncertainty renders decision priorities unclear. Probabilistic methods to quantify uncertainty can further inform implementation research efforts through formal Value of Information analyses [12, 66–68]. If the overall goal is to reduce the uncertainty in a decision, then these analyses can be used to place a specific value on future research studies that can provide more information (and thus reduce decision uncertainty) [12, 66–68].

All case studies integrated their results. Cruden created “summary scores” for each of the three EBPs assessed [37]. These summary scores were calculated for each stakeholder, as a weighted sum of intervention scores, using weights that stakeholders modified for their own context [37]. Zimmerman reported visual trends in EBP reach (sessions scheduled, completed) under different implementation plans and used sensitivity analyses and stakeholder review to validate their model [38]. Other implementation planning work, also in the VA though focused on stroke, has also used system dynamics modeling and reported extensive sensitivity and uncertainty analyses [69, 70]. Finally, Hassmiller Lich presented a visual that depicted how the cost of each intervention and the life years up-to-date were related and discussed which of the interventions were likely to be the most cost-effective, as well as the 10-year investment required [39]. While this model was probabilistic (meaning that each replication would result in a different answer, based on initial simulated values), conducting a full probabilistic analysis was not feasible given the size of the model [39]. Thus, they report mean values of outcomes over 10 large, full population replications [39].

## Discussion

Decision analysis provides structure to guide users to clearly articulate and agree upon objectives (e.g., improve outcomes, decrease costs, reduce inequities), uncover diverse decision alternatives, consider the strengths and weaknesses of each alternative in context, and examine potential trade-offs in objectives under each alternative [17]. These steps are a clear fit with and can add rigor to implementation planning, where implementors typically need to compare different implementation alternatives, understand the potential consequences of those alternatives, and decide what is suitable for their context. Importantly, decision analysis does not prescribe which kinds of consequences should be examined or what is right for a given context. The choice of what consequences to assess, how to value those impacts, and how the valuation leads to a decision is always context-specific and should incorporate the preferences and values of all and often diverse stakeholders [17, 33, 61, 71]. Additionally, while many of the individual components of decision analysis may be familiar to implementation scientists (e.g., engaging stakeholders, selecting candidate implementation strategies), we believe it is valuable to situate these component pieces within a broader decision analysis framework.

All pieces of the PROACTIVE framework may not always be needed, and the component pieces within the framework may not proceed linearly. After the discussion of objectives, stakeholders might need to reevaluate the problem at hand. Sometimes, it may be that refining the problem definition and objectives provokes enough discussion to uncover a clear decision. Other times, the problem may be clear, and more effort is needed to assess the potential effects under each alternative or understand where major uncertainties are. This drives home that while the process may be constructed around coming to a decision, learning and insight gained throughout the decision analysis process can often be just as valuable [17, 71], and once a process is completed for a given decision problem, the investment made can support subsequent decision-making.

### Future directions

The value of applying decision analytic approaches to implementation planning has been suggested in other work, though these methods remain underutilized [12, 37, 38, 60, 71]. To advance the use of decision analysis in implementation planning and research, we propose key areas for future research and practice based on issues considered in this paper and encourage discussion on how these suggestions can be prioritized by the field.

#### Embrace model thinking

In addition to the complexity that can arise in the process of making decisions, complexity also exists within systems where implementation occurs (time delays in seeing the impacts of action, feedback loops, nonlinear relationships) [52–56]. Some modeling methods are designed to incorporate this. However, any model will, by definition, be a simplification of reality and require setting boundaries around what is included. Using initial models that represent our current, best understanding of problems and iteratively revising them as our knowledge of the system and problem grows is crucial [22, 71]. Models can be leveraged to make assumptions transparent or further improve our understanding of complex problems [71]. For example, a small model could help identify where we need more data or knowledge—what parameters or structures are uncertain? Why are outcomes so sensitive to a specific component of the system? What happens when we consider different perspectives or constraints, or involve those with other expertise? As we learn from answers to these questions, we can expand the initial model and continue to use it to improve outcomes in complex and changing contexts.

#### Build the business case for decision analysis approaches

Publications and reports that describe if and how upfront investment planning improves downstream outcomes can help build momentum for future applications of decision analysis. It is possible that decision analysis could reduce long-term costs by helping implementors choose interventions and implementation strategies with the greatest chance of success in a specific context. However, we need to test this assumption in our research and invest in research that evaluates the impact of decision analysis on decision-making and downstream outcomes [72].

#### Identify when, how, and for whom decision analytic approaches are useful

As others engage in decision analysis, detailed publications of nuances, objectives, and lessons learned through the process can improve our understanding of how and when pieces of decision analysis are best employed. The application of larger modeling efforts within a decision analysis may be most helpful when many actors are involved or when large sums of money are funds at stake, like in large health systems or at the state or federal level (e.g., Hassmiller Lich et al., Table 1 [39]). At lower “levels” like in communities or clinics, where even more might be at stake given tighter budgets, investigating how decision analytic approaches can reasonably be deployed should be prioritized. For example, work could evaluate how a structured decision analysis process or a small model that captures the major complexities support planning efforts (e.g., Cruden et al., Table 1 [37]).

#### Improve reporting and transparency of cost data

Decision analysis approaches require estimates from published literature, drawing on, for example, studies of implementation effectiveness and work evaluating the costs of implementation strategies. Thoughtfully considering how prior literature can inform future decision-making in different contexts is thus core to any decision analysis, and all inputs used throughout a decision analysis should be interrogated and justified [73]. One major area of current focus with implementation science that is often missing detail is the costs of implementation [74–77]. Recent publications by the “Economics and Cost” action group of the Consortium for Cancer Implementation Science have set out definitions, guidance, methods, and best practices for understanding the costs of implementation and conducting economic evaluations in implementation science [36, 74, 77–80] and complement ongoing work in the field [6, 8, 12, 14, 61, 75, 76, 81–84].

To address gaps in reporting of cost data, scholars have called for consistent and detailed reporting on intervention costs, intervention adaptations, applied implementation strategies, and accrued implementation costs [6, 9, 40, 61, 85]. Refining existing reporting guidelines to help authors standardize what is reported could greatly improve the likelihood that published work could inform decision analytic approaches in the future. For example, the Consolidated Health Economic Evaluation Reporting Standards (CHEERS) checklist guides the reporting of economic evaluation [86, 87]. This checklist could be modified to prompt detailed reporting on implementation processes, including actors, timing, purpose, and costs related to each implementation decision or action—quite similar to previous calls for standardized reporting [6] and existing recommendations for the reporting of implementation strategies [40].

In addition, efforts should be made to improve the transparency of cost data and data sharing. Cost data is often sensitive or proprietary. Presenting the implementation costs associated with specific sites may allow sites to be identified, raising questions about confidentiality in research. However, incorporating costs into decision analysis depends on transparency and willingness to share data and processes, and the field should consider how to address these issues.

**Fig. 2:**
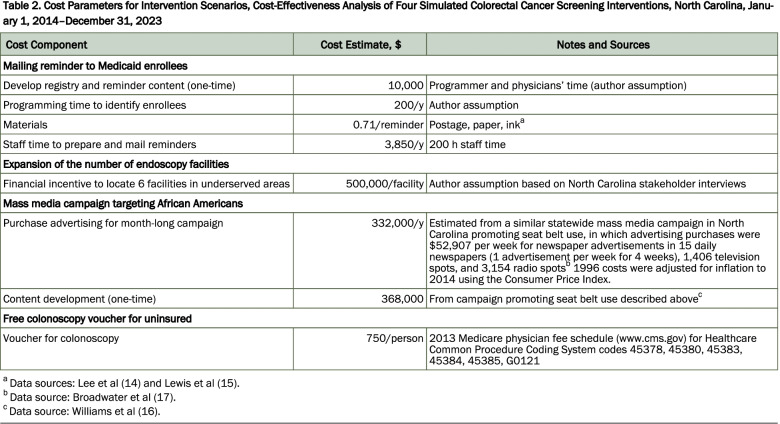
Illustrative example of costs reported to facilitate transparency and inform decision analytic approaches in other contexts. *Notes*: Reproduced Table 2 from Hassmiller Lich et al. [39]. This figure shows the cost estimate inputs required for decision analysis approaches and the variety of potential sources for estimates

As an example of how we envision reporting and transparency considerations might be operationalized in future published work, Hassmiller Lich et al. specified the different cost components of each intervention they considered, whether costs were one time or recurring, and specific notes on data sources (Fig. 2) [39]. This makes the paper of broader use even though the model was specific to North Carolina; future research can build on this work to better understand the components of costs that might be incurred if similar interventions were implemented in different contexts, even if the specific values might differ.

#### Increase collaborative opportunities and training

Demonstrations of decision analysis processes in the peer-reviewed literature can help bolster the evidence base for others to learn from. A focus on implementation planning and decision analysis could also be integrated into existing training programs. In situations where more expertise is needed, decision analysis experts should be engaged (perhaps specifically to help facilitate identifying a clear problem, modeling potential consequences using simulation approaches, or assessing preferences). These experts are often in disciplines like systems science, operations research, health services research, health policy, or explicit decision science fields. Many of these experts are trained to collaborate on interdisciplinary teams and can be complements to collaborators with a deep understanding of implementation complexities and subject matter expertise.

## Conclusions

An increased attention to decision analysis can provide a dual benefit to the field of implementation science by lending structure to implementation planning and helping to uncover innovative directions for future research. A key strength of decision analysis is its flexibility to be used in the way that is best suited to a given context, and we hypothesize even a simple analysis executed thoughtfully can be powerful. We encourage implementation scientists to use decision analysis principles in their own work and report on their experiences to help drive the field forward and contribute to better implementation outcomes.

## Data Availability

Not applicable.

## Abbreviations

CFIR: Consolidated Framework for Implementation Research
EBP: Evidence-based program
CHEERS: Consolidated Health Economic Evaluation Reporting Standards
